# Effect of Different Fertilization on Soil Fertility, Biological Activity, and Maize Yield in the Albic Soil Area of China

**DOI:** 10.3390/plants14050810

**Published:** 2025-03-05

**Authors:** Xingzhu Ma, Yue Zhao, Yu Zheng, Lingli Wang, Yulan Zhang, Yi Sun, Jinghong Ji, Xiaoyu Hao, Shuangquan Liu, Nan Sun

**Affiliations:** 1 Heilongjiang Academy of Agricultural Sciences, Harbin 150086, China; 2 Heilongjiang Academy of Black Soil Conservation and Utilization, Harbin 150086, China; 3 Key Laboratory of Black Soil Protection and Utilization, Ministry of Agriculture and Rural Areas, Harbin 150086, China; 4 Institute of Applied Ecology, Chinese Academy of Sciences, Shenyang 110016, China; 5 Institute of Agricultural Resources and Regional Planning, Chinese Academy of Agricultural Sciences, Beijing 100081, China; sunnan@caas.cn

**Keywords:** optimized fertilization, albic soil, soil fertility, soil biological activity, maize yield

## Abstract

Fertilization is a key management practice for maintaining or improving soil fertility and ensuring grain yield in agro-ecosystems. Nevertheless, as a low-yield soil, how fertilization strategies impact the status of albic soil physical and chemical properties, biological activity, and crop yield are poorly understood. Through a two-year positioning experiment, the albic soil fertility characteristics (physical, chemical, and biological) and changes in maize yield under different fertilization were studied. Three treatments were established: (1) conventional fertilization (chemical fertilizer) (T1), (2) optimized fertilization 1 (low amount of organic fertilizer + chemical fertilizer) (T2), and (3) optimized fertilization 2 (high amount of organic fertilizer + chemical fertilizer) (T3). The results indicated that, compared with T1, the soil bulk density of T2 and T3 treatments decreased, the average solid phase ratio of soil decreased by 8.2%, and the average liquid and gas phase ratios increased by 7.2% and 10.2%, respectively. The soil organic matter (SOM) and soil organic carbon storage (SOCS) under treatment of optimized fertilization were significantly higher than under T1, with an average increase of 10.1% for SOM and 8.8% for SOCS, respectively. T3 significantly increased the contents of alkali-hydrolyzable nitrogen, available phosphorus, and available potassium, while different fertilizations had little effect on soil pH. T2 and T3 significantly increased activities of soil urease, sucrase, phosphatase, and catalase, with an average increase of 33.7%, 56.9%, 32.0%, and 6.7%, respectively. The numbers of soil bacteria and actinomycetes under T3 increased significantly by 30.2% and 22.0% compared to T1, while the number of fungi decreased by 6.7%. The total number of soil microorganisms increased significantly by 29.0% of T3, and the proportion of soil bacteria to the total number of microorganisms increased, while the proportion of fungi and actinomycetes decreased. The maize yield of T3 was significantly higher than under other treatments, with an increase of 2368.5 kg/ha compared to T1. Correlation analysis showed that the contents of available nutrients and organic matter, the numbers of soil bacteria and actinomycetes, and the activities of soil urease and phosphatase had the most significant impact on maize yield. The optimized fertilization, which was the organic fertilizer combined with chemical fertilizer, can improve the physical properties of albic soil, increase soil organic matter content, organic carbon storage, available nutrient content, and soil biological activity, also for maize yield. Therefore, the optimized fertilization in albic soil of Northeast China is a promising and important management option for improved soil quality and grain yield. This work provides a theoretical basis and technical reference for efficient fertilization.

## 1. Introduction

Albic soil is one of the cultivated soils in Northeast China, mainly distributed in the eastern part of Heilongjiang and Jilin provinces, with a total area of 5.272 million hectares. Among these, the cultivated land albic soil area is 1.6668 million hectares, accounting for 31.6% of the total albic soil area [1]. The Sanjiang Plain, which is located in the eastern part of Heilongjiang province, is a concentrated distribution area of albic soil, with a total area of 2.23 × 10^6^ ha [2,3]. At present, albic soil is classified as a regional low-yield soil mainly due to its physical and chemical characteristics. Firstly, the black soil layer of albic soil is thin and the total nutrient storage is low; the thickness of the black soil layer and sub-layer of the albic layer on the surface are all about 20 cm [4,5]. Secondly, the poor physical properties and high bulk density of the albic layer lead to poor water and gas transmission between the upper and lower soil layers, which seriously hinders crop growth [6], resulting in low and unstable crop yields. Research on the improvement of albic soil, which focused on tillage techniques such as deep loosening, deep plowing, and subsoil mixing, began in the 1960s and 1970s in China [7,8]. Subsequently, organic material improvement was carried out successively, including straw return [9], use of organic fertilizer [2], and addition of biochar [10,11]. In terms of mechanical improvement of albic soil, research has been conducted on the application of subsoil interval mixed layer plowing [12] and subsoil mixing ploughing with fertilizer [13]. Many improvements of albic soil mainly focused on techniques and machinery in the past, while there was relatively little research on optimizing fertilization and fertilizer application. Research on agricultural techniques such as fertilization and tillage in other regions has shown that fertilization has a direct effect in increasing soil nutrient content, affecting soil biological activity, and indirectly promoting nutrient transformation in the soil [14]. For example, organic fertilizer combined with chemical fertilizers can increase the number of bacteria, fungi, and actinomycetes in purple soil in China [15], as well as the activity of soil enzymes and nutrient contents [16]. At the same time, it can increase the yield of crops such as wheat [17], soybeans [2], and maize [10].

Conducting research on optimizing fertilization to alleviate the resource and environmental pressures caused by unsustainable fertilization is of great significance for improving agricultural production capacity in albic soil areas and promoting healthy and sustainable agricultural development in China. Therefore, we applied different fertilizations in the albic soil area, and tested and analyzed soil physical and chemical indicators, soil biological activity (enzyme activity, microbial quantity), and maize yield, aiming to reveal the effects of different fertilizations on soil fertility and soil biological activity, explore the changes of soil fertility and crop yield in low-yield soil areas, and lay a theoretical foundation for rational fertilization in the albic soil region in China, which will provide technical support for stabilizing and improving grain yield.

## 2. Materials and Methods

### 2.1. Study Site

This experiment was conducted from 2023 to 2024, and the experimental area is located in the 852 Farm Management Zone (46°17′ N, 132°44′ E) of Shuangyashan City, Heilongjiang Province, China. This area belongs to the cold temperate continental monsoon climate, the annual average temperature is 3.2 °C, the annual average precipitation is 548 mm, the average accumulated temperature above 10 °C is 2700 °C, and the annual average frost-free period is 140~150 days [18]. The contents of total organic matter, total nitrogen, total phosphorus, and total potassium of the soil when the experiment started were 37.3, 5.81, 1.79, and 0.68 g/kg, respectively; the contents of alkaline nitrogen, available phosphorus, and available potassium were 165.3, 16.5, and 89.0 mg/kg, respectively; and the pH was 5.89.

### 2.2. Experimental Design

We made use of a field experiment, with each plot covering an area of 390 m^2^ and three replicates. Three fertilization treatments were set up: conventional fertilization (chemical fertilizer) (T1), optimized fertilization 1 (low amount of organic fertilizer + chemical fertilizer) (T2), and optimized fertilization 2 (high amount of organic fertilizer + chemical fertilizer) (T3). The conventional fertilization treatment uses formula fertilizer (nutrient content of N-P_2_O_5_-K_2_O is 20-20-16) with the application rate of 525 kg/ha, and topdressing uses urea with the application rate 270 kg/ha. The organic fertilizer belonged to a type of fermentation tank organic fertilizer in the form of powder with nutrient content (N + P_2_O_5_ + K_2_O) ≧ 4%, and organic matter content ≧30%. The amount of high organic fertilizer was 3000 kg/ha, and the amount of low organic fertilizer was 1500 kg/ha; all fertilizers, except for urea as topdressing, were used as base fertilizers for one-time application to a depth of 15–20 cm in the soil during spring. The tested crop was maize (*Zea mays* L.) of the variety Denia 3, sown in May and harvested in October, with a planting density of 70,000 plants/ha during the years 2023 and 2024. The ridge width for planting maize is 130 cm. There was no irrigation during the crop growth period, and other management measures were consistent with local conventional production.

### 2.3. Soil Sampling, Measzurement, and Crop Yield Measurement

Surface soils (0–20 cm) were collected after maize harvest in October 2024. Five soil cores were taken randomly as one sample in each plot, mixed thoroughly, and then pooled in a sterile plastic bag on ice for transportation to the laboratory. The samples were sieved through a 2.0 mm sieve and divided into two portions: the first portion was preserved at 4 °C for the determination of soil enzyme activities and microbial quantities, the second portion was air-dried for soil chemical properties measurement.

Soil bulk density in the 0–20 cm soil layer was measured by the cutting ring method. The ratios of soil solid, liquid, and gas were determined using a soil three-phase analyzer (DIK-1150, Daiki, Japan). The alkaline nitrogen, available phosphorus, and available potassium in the soil were determined by alkaline diffusion, Olsen, and ammonium acetate flame photometry methods, respectively. The soil pH was determined by the potentiometric method, as described in a previous study [19]. Organic matter content was determined by the method of potassium dichromate volumetry [20]. The numbers of bacteria, fungi, and actinomycetes were determined using the method of dilution plate counting and activities of soil urease, phosphatase, sucrase, and catalase were determined by the indophenol blue colorimetric method, disodium phenyl phosphate colorimetric method, sodium thiosulfate titration method, and potassium permanganate titration method, respectively; the methods were described in a previous study [19].

Maize yield was determined after harvest on sub-plots of 6.5 m^2^ with three replicates, and the actual grain yields were calculated based on a moisture content of 14%.

The calculation of soil organic carbon storage is as follows [21]:

SOCstock = (SOC i × BD × H i) × 0.1(1)

where SOCstock is the soil organic carbon storage at a certain depth (t/ha); SOC i is the organic carbon concentration of the i-th layer of soil (g/kg); BD is the bulk density of the i-th layer of soil (g/cm^3^); and H i is the thickness of the i-th layer of soil (cm), with a unit conversion coefficient of 0.1.

### 2.4. Statistical Analysis

Data statistics and analysis were conducted using the software Excel 2016 and SPSS 19.0; the LSD method was used to conduct variance analysis to test the significance of differences between different treatments. The Pearson method was used for correlation analysis, and Origin 2019 was used for the correlation graph. SigmaPlot 10.0 was used for other plotting purposes.

## 3. Results

### 3.1. Physical and Chemical Properties of Albic Soil

#### 3.1.1. Changes of Soil Three-Phase Ratio and Soil Bulk Density

Soil three-phase ratio and soil bulk density under different fertilization are shown in Table 1. There were no significant differences in soil three-phase ratio and bulk density among different treatments; the three-phase ratio of soil in all treatments showed the following trend: solid phase > liquid phase > gas phase. The conventional fertilization (T1) had the highest soil solid phase percentage (51.85%), and the liquid phase and gas phase percentages were both lower. Compared with T1, the optimized fertilization (T2 and T3) could reduce the soil solid phase percentage (7.3% and 9.2%, respectively), and increase the liquid phase and gas phase percentages. The changes of soil bulk density under different treatments followed T1 > T3 > T2, overall; the soil bulk density of T2 and T3 was slightly lower than T1, with an average decrease of 1.2%.

#### 3.1.2. Soil Organic Matter Content and Soil Organic Carbon Storage

**Figure 1:**
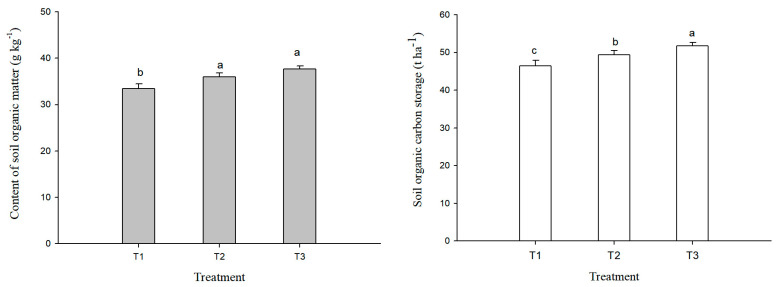
Organic matter content and organic carbon storage of albic soil under different fertilization treatments. T1: conventional fertilization (chemical fertilizer), T2: optimized fertilization 1 (low amount of organic fertilizer + chemical fertilizer), T3: optimized fertilization 2 (high amount of organic fertilizer + chemical fertilizer). Different lowercase letters indicate significant difference among treatments at the *p* < 0.05 level.

There was a significant difference in contents of albic soil organic matter under different treatments (Figure 1). The optimized fertilization with organic fertilizer combined with chemical fertilizer had a significantly higher soil organic matter content than the conventional fertilization, in the order of T3 > T2 > T1. Compared with T1, the soil organic matter content of T2 and T3 increased by 2.57 g/kg and 4.22 g/kg, respectively, with an average increase of 10.1%; T3 increased by 12.6%. The changes of albic soil organic carbon storage under different treatments were consistent with the changes in soil organic matter content. The optimized fertilization significantly increased the soil organic carbon storage compared to conventional fertilization; the soil organic carbon storage of T3 was significantly higher than those in T2 and T1. Compared with T1, the soil organic carbon storage in T3 and T2 increased by 5.27 t/ha and 2.92 t/ha, respectively.

#### 3.1.3. Soil Nutrient Contents

The soil chemical nutrient contents (alkaline nitrogen, available phosphorus, and available potassium) under different fertilization are shown in Table 2. The results showed that the differences in soil alkaline nitrogen, available phosphorus, and available potassium content under different treatments were significant, while differences in soil pH were not significant. The combination of organic fertilizer and chemical fertilizer treatment increased soil available nutrient contents, in the order T3 > T2 > T1. The changes in soil available phosphorus and available potassium content were greater than those in soil alkaline nitrogen. Compared with T1, T2 and T3 increased soil alkaline nitrogen content by 4.3% and 8.1%, soil available phosphorus content by 39.0% and 59.0%, and soil available potassium content by 27.8% and 43.5%, respectively. The changes in soil pH values were relatively small under different treatments, and the differences were not significant.

### 3.2. Biological Activity of Albic Soil

#### 3.2.1. Soil Enzyme Activity

**Figure 2:**
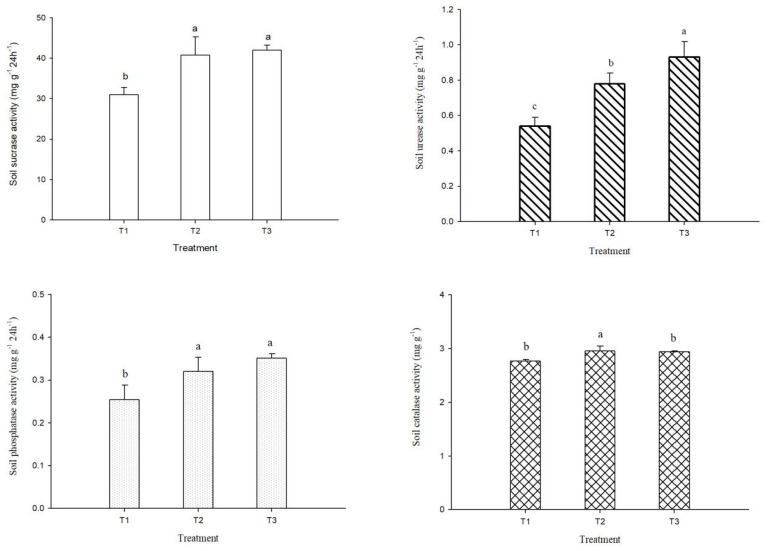
Enzyme activities of albic soil under different fertilization treatments. T1: conventional fertilization (chemical fertilizer), T2: optimized fertilization 1 (low amount of organic fertilizer + chemical fertilizer), T3: optimized fertilization 2 (high amount of organic fertilizer + chemical fertilizer). Different lowercase letters indicate significant difference among treatments at the *p* < 0.05 level.

Figure 2 shows the activities of soil sucrase, urease, phosphatase, and catalase under different fertilization treatments. Different fertilization treatments had an important influence on soil enzyme activity. T2 and T3 significantly increased soil sucrase activity, but there was no significant difference between them. Compared with T1, T2 and T3 increased soil sucrase activity by 31.8% and 35.5%, respectively. The order of soil urease activity was T3 > T2 > T1, and the differences between treatments were significant. Compared with conventional fertilization (T1), T2 and T3 increased soil urease activity by 0.24 mg·g^−1^·24 h^−1^ and 0.39 mg·g^−1^·24 h^−1^, respectively, with an average increase of 56.9%. The changes of soil phosphatase activity were consistent with those of soil sucrase, i.e., the activities under the optimized fertilization was significantly higher than under the conventional fertilization. Compared with T1, the soil phosphatase activity in T2 and T3 increased by 25.8% and 38.2%, respectively. The changes in soil catalase activity were basically consistent with those for the other three enzymes. The soil catalase activity in T2 and T3 was higher than that in T1, with an average increase of 6.7%, and the activity in the T2 treatment was significantly higher than in the T1 and T3 treatments.

#### 3.2.2. Soil Microbial Quantities

(1) Quantities of the three major soil microbial communities

**Figure 3:**
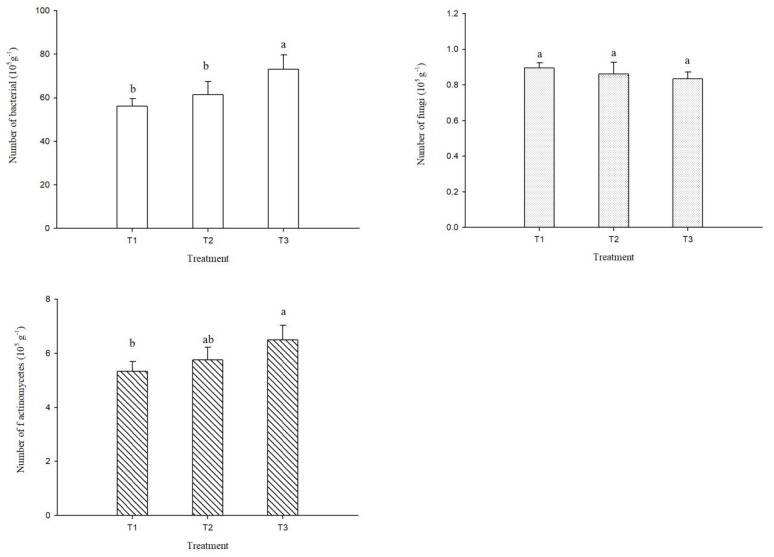
Numbers of bacteria, fungi, and actinomycetes of albic soil under different fertilization treatments. T1: conventional fertilization (chemical fertilizer), T2: optimized fertilization 1 (low amount of organic fertilizer + chemical fertilizer), T3: optimized fertilization 2 (high amount of organic fertilizer + chemical fertilizer). Different lowercase letters indicate significant difference among treatments at the *p* < 0.05 level.

The numbers of bacteria, fungi, and actinomycetes in albic soil under different treatments are shown in Figure 3. Optimizing fertilization increased the number of soil bacteria; compared with T1, the number of soil bacteria in T2 and T3 increased by 9.4% and 30.2%, respectively. The number of soil bacteria in T3 was significantly higher than that in T1 and T2. The trend for soil fungal quantity changes was different from that for bacteria. T1 had the highest soil fungal quantity, followed by T2, while T3 was lowest. The differences in soil fungal quantity under different treatments did not reach a significant level. The optimized fertilization reduced the average soil fungal quantity by 5.3%. The number of soil actinomycetes changed in the same way to that of bacteria; the optimized fertilization was higher than conventional fertilization. Compared with T1, the average increase in soil actinomycete count in T2 and T3 was 14.9%, and the number of soil actinomycetes in T3 was significantly higher than in T1.

(2) Total numbers and proportions of soil microorganisms

Results in Table 3 indicate that the optimized fertilization treatment had a higher total number of soil microorganisms than the conventional fertilization treatment. The total number of soil microorganisms in T3 was significantly higher than in T1 and T2; compared with T1, T3 increased the total number of soil microorganisms by 29.0%. The proportion of soil bacteria to the total number of microorganisms under different fertilization treatments followed the order T3 > T2 > T1, and there was no significant difference among treatments. The increase in the proportion of soil bacteria to the total number of microorganisms in the optimized fertilization treatment was relatively small (0.3~1.0%). Compared with T1, the proportion of soil fungi in T2 and T3 decreased by 11.9% and 27.4%, respectively, with T3 significantly lower than T1. There was no significant difference in the proportion of soil actinomycetes among different fertilization treatments.

### 3.3. Yield of Maize and Its Relationship with Soil Properties

#### 3.3.1. Yield of Maize

**Figure 4:**
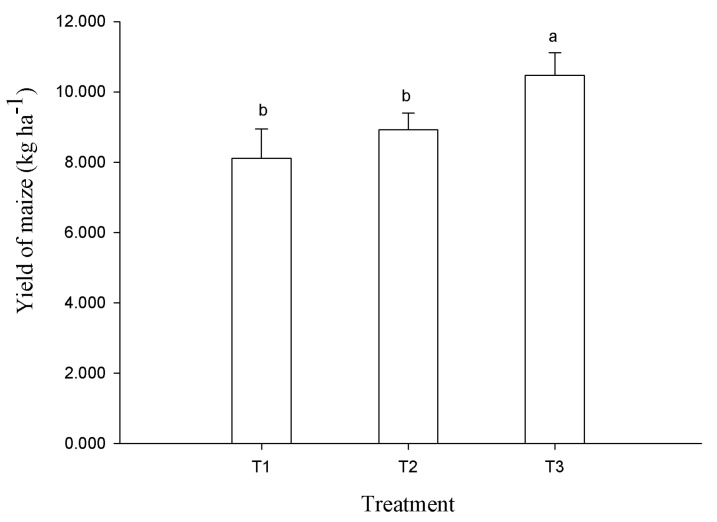
Yield of maize under different fertilization treatments. T1: conventional fertilization (chemical fertilizer), T2: optimized fertilization 1 (low amount of organic fertilizer + chemical fertilizer), T3: optimized fertilization 2 (high amount of organic fertilizer + chemical fertilizer). Different lowercase letters indicate significant difference among treatments at the *p* < 0.05 level.

The order of maize yield under different fertilization treatments was as follows: T3 > T2 > T1 (Figure 4), which indicated that the optimized fertilization (T2 and T3) gave a higher overall yield than the conventional fertilization (T1). Compared with T1, the average yield of maize under the T2 and T3 treatments increased by 19.7%, with the T3 treatment significantly improving maize yield.

#### 3.3.2. Relationships Between Maize Yield and Soil Chemical, Physical, and Biological Activity Indicators

**Figure 5:**
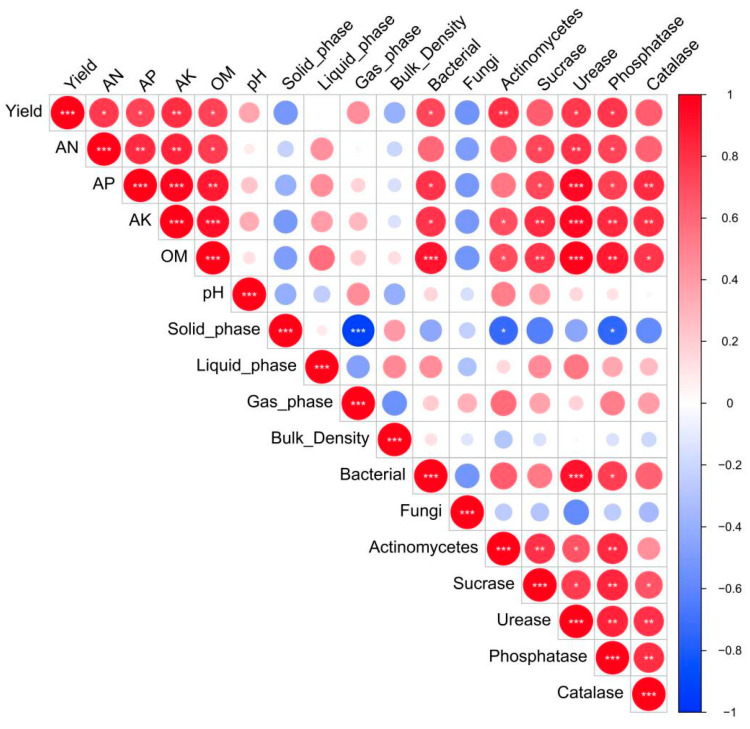
Correlation analysis between maize yield, physical and chemical characteristics, and biological activity of albic soil. Red indicates positive correlation, blue indicates negative correlation. The darker the color, the larger the circle, the larger the correlation coefficient between the two indicators. If the two indicators are not correlated, no circle is displayed. AN: alkaline nitrogen; AP: available phosphorus; AK: available potassium; OM: organic matter. *, ** and ***: statistical significance at *p* < 0.05, *p* < 0.01 and *p* < 0.001, respectively.

Soil physical and chemical properties, and biological activity, directly affect soil fertility levels, which affects crop yields indirectly. Figure 5 shows the correlation analysis between maize yield and physical, chemical, and biological activity indicators of albic soil. Results indicated that relationships between maize yield and soil physical indicators under different treatments were not significant, but that there were negative relationships between maize yield and the soil solid phase ratio and soil bulk density. There were positive and significant relationships between maize yield and soil chemical indicators, including soil organic matter and available nutrient contents, but not for soil pH. The yield of maize was positively and significantly correlated with the number of soil bacteria and actinomycetes, and negatively, but not significantly, correlated with the number of fungi. There was a positive correlation with soil enzyme activities, with a positive and significant relationship with soil urease and phosphatase activity. In addition, there was a significant and positive correlation between soil chemical indicators and soil enzyme activities, while there was no significant correlation between soil physical indicators and soil microbial quantities. In summary, maize yield was closely related to soil chemical indicators, enzyme activities, and microbial quantities. A higher level of soil fertility and soil biological activity would thus increase crop yield.

## 4. Discussion

### 4.1. Effect of Fertilization on Soil Physical Properties

Albic soil has some key problems such as its lower soil available water content and excessive soil hardness, etc. The occurrence of the albic soil layer was shallow, resulting in a shallow effective soil layer in the plow layer [5,6]. Therefore, improving the albic soil layer has become the main goal for the improvement of this soil. This study mainly focused on the utilization and improvement of albic soil from the perspective of fertilization. The soil bulk density under the optimized fertilization (T2 and T3) was slightly lower than that under the conventional fertilization (chemical fertilizer alone) in the current study (Table 1), which might be related to the application of organic fertilizers increasing soil porosity and reducing soil compaction, resulting in a decrease in soil bulk density and effective improvement of soil physical properties [22]. As this study was only conducted for two years, the differences between the treatments did not reach a significant level, and further, more long-term, research is needed to detect possible significant changes in the future. The three-phase relationship of soil refers to the quantitative relationship between the solid, liquid, and gas phases in the soil, reflecting the amount and proportion, as well as the energy state, structure, and physical properties of each phase [23]. The trend for soil three-phase ratio changes under different treatments was basically consistent with that for soil bulk density in the current study, and there were no significant differences between treatments. However, the overall three-phase ratio showed a decrease in the soil solid phase percentage and an increase in the liquid and gas phase percentage after applying organic fertilizer combined with chemical fertilizer (Table 1), which indicated that the use of organic fertilizer reduced the soil solid phase percentage, increased the liquid and gas phase percentages, and promoted soil permeability. Addition of organic materials can improve the physical structure of the plow layer soil, enhance the water and gas supply capacity of the soil to crops, and create a more suitable soil structure for crop growth [18].

### 4.2. Effect of Fertilization on Soil Chemical Properties

Previous studies have shown that the combination of organic and chemical fertilizers can improve soil fertility and play an important role in increasing soil organic carbon accumulation and optimizing the soil environment [24]. Soil organic matter content and organic carbon storage under optimized fertilization were significantly higher than those under conventional fertilization in the current study (Figure 1), which was consistent with previous studies in which it was reported that the application of organic–inorganic fertilizers increased soil organic carbon content, and a higher amount of organic material input resulted in a higher content of soil organic carbon [25]. Treatment with a combination of organic fertilizer and chemical fertilizer may lead to the addition of a large amount of nutrients into the soil, providing a suitable growth environment for microorganisms and creating conditions conducive to soil organic carbon fixation. In addition, factors such as carbon input, carbon output by crop carried, and soil respiration also affect soil organic matter content and carbon storage [26]. Research on the improvement of albic soil with different types of organic fertilizers has shown that organic fertilizers promote the conversion of fulvic acid to humic acid, which is beneficial for increasing the content of humus [27]. Therefore, external carbon input and a certain carbon sequestration capacity are significant in improving the quality of albic soil and increasing crop yield.

There were significant differences in the contents of soil alkali-hydrolyzable nitrogen, available phosphorus, and available potassium among different treatments in the current study (Table 2). Maize has a high demand for nutrients, especially nitrogen. Studies have shown that about 35% of nitrogen in mature maize plants comes from fertilizer nitrogen, and about 65% comes from soil nitrogen, indicating that soil is still the main source of nitrogen supply for maize [28]. Shen et al. (2023) studied organic fertilizer substitutions and demonstrated that different substitution rates of organic fertilizer could improve the soil fertility (27.5–35.0%), compared with treatment with chemical fertilizer alone [29]. Treatment with slow-release nitrogen fertilizer combined with organic fertilizer could significantly increase the available phosphorus and potassium contents in paddy soil, while there was little effect on soil pH [30]. Results from these studies were consistent with the current study; we found that adding organic fertilizers can maintain a high overall level of available nutrient content in the plow layer soil, which helps to fertilize the soil. The combination of chemical fertilizers and organic fertilizers can increase the content and availability of available nutrients in the soil, and some available nutrients in organic fertilizers accelerate soil microbial activity directly or indirectly, thereby providing more available nutrients [31].

### 4.3. Effect of Fertilization on Soil Biological Activity

Compared with T1, optimizing fertilization treatments (T2 and T3) significantly increased the activities of soil sucrase, urease, acid phosphatase, and catalase in this study, and the activities under T3 were higher than under T2 (Figure 2), which was consistent with the research results for soil enzyme activity with other crops such as maize and wheat treated with organic fertilizer combined with chemical fertilizer [17,32]. Changes in soil enzyme activities may be related to the application of organic fertilizer, which has the effect of improving soil structure; organic fertilizer can promote the formation of stable complexes between soil enzymes, organic matter, and soil aggregates, thereby enhancing soil enzyme activity [33]. In addition, the organic matter in organic fertilizers might provide sufficient carbon sources for enzyme-producing microorganisms [34]. The T2 and T3 treatments in the current study significantly increased the number of soil bacteria and actinomycetes (Figure 3), which is consistent with findings on soil microbial populations in crops such as maize and rice, i.e., that the application of organic fertilizer combined with chemical fertilizer can significantly increase the numbers of soil bacteria and actinomycetes and reduce the number of fungi [35,36]. This may be related to a high C/N ratio of organic fertilizer, which is useful after application for enhancing the activity of soil microorganisms [37]. Application of organic fertilizer increased the total number of the three major soil microbial communities, also increasing the proportion of soil bacteria and decreasing the proportions of fungi and actinomycetes (Table 3). This is mainly because organic fertilizer and its decomposition can increase the types and quantities of soil carbon sources, providing sufficient substrates for microbial metabolic activities. This process stimulates soil microbial activity and directly affects soil nutrient conversion efficiency, thereby achieving soil nutrient activation, changing soil microbial diversity and biomass composition [38,39].

### 4.4. Effect of Fertilization on Crop Yield

Previous studies have shown that different organic materials being returned to the field and mechanical soil modification methods affect crop yields [12,18]. This study focuses on the relationship between fertilization methods and crop yield. The combination of organic fertilizer and chemical fertilizer could significantly increase maize yield, with an average increase of 19.7% for T2 and T3 (Figure 4). Previous results are consistent with the current study; for example, organic fertilizer and its combination with chemical fertilizer increased the yield of maize in albic soil and alkaline farmland under straw return conditions [40,41], increased the dry matter content of sorghum in a positioning experiment [22], and increased the grain yield of wheat and corn in loess soil [42]. In addition, the sustainability index values for crop yield under a combination of organic and chemical fertilizer were higher than under chemical fertilizer treatment alone in the black soil area of China [43]. Compared with the application of chemical fertilizer alone, organic fertilizer instead of chemical fertilizer at rates of 20% and 30% could significantly increase wheat grain yield and straw yield [44]. Different types of organic fertilizers combined with chemical fertilizers have been shown to increase crop yield, including granular organic fertilizer with pig manure as the substrate and slow release urea [30], cow manure with compound fertilizer [22], potassium fulvic organic fertilizer [44], pig manure fermented organic fertilizer [45], and commercial organic fertilizer made of Ru cake, soybean meal, and fried pancake [46]. The crop yield increase after the application of organic fertilizer was mainly due to its role in supplementing and balancing soil nutrients; the application of organic fertilizer was beneficial for the growth and extension of crop roots, which not only improved soil physical properties but also promoted crop growth, thereby increasing crop yield [22].

### 4.5. Effects of Fertilization on the Relationship Between Crop Yield, Soil Fertility, and Biological Activity

Soil physical and chemical properties, together with biological activity, affect crop growth and yield through their indirect and direct effects, respectively. The use of subsoil interval mixed layer technology to improve albic soil reduced soil hardness and bulk density, increased contents of available nutrients and total nutrients in the soil 20–40 cm layer, increased soil pH and organic matter content, and ultimately increased soybean yield by about 5% and corn yield by about 20% [47]. Organic material return could significantly increase the organic matter content in the plow layer (0–35 cm) of albic soil under deep plowing; the mixing of organic materials in the 35 cm layer significantly increased maize yield, and the content of organic matter was highly positively correlated with maize yield [40]. The results on fertilization in saline alkali farmland indicated that there is a highly significant positive correlation between maize yield and contents of organic matter, total nitrogen, and available phosphorus, and a significant negative correlation with pH [41]. In addition, reasonable organic fertilization had significant effects on distribution characteristics of soil organic carbon components, improving soil comprehensive fertility and soil carbon storage [48]. The correlations between maize yield and soil physical and chemical properties in the current study were consistent with the previous research results mentioned above. Meanwhile, maize yield was negatively correlated with the number of soil fungi, but positively correlated with other biological activity indicators (soil enzyme activity, numbers of soil bacteria, and actinomycetes), and showed significant correlation with some indicators (Figure 5), indicating that the enhancement of soil biological activity is beneficial for increasing crop yield. This might be due to soil biological activity indicators such as soil enzyme activity and soil microbial quantity being closely related to soil nutrients, and being very sensitive to changes in soil quality and health [49]. Therefore, optimizing fertilization can help improve soil physical structure, enhance the soil fertility level, and increase soil biological activity, thereby increasing crop yield.

## 5. Conclusions

Compared to conventional fertilization, optimizing fertilization by combining organic and chemical fertilizers can comprehensively improve and enhance the fertility of albic soil. Firstly, optimizing fertilization reduced the bulk density and solid phase ratio of the topsoil layer, increased the liquid and gas phase percentages, and created favorable soil physical conditions. Secondly, optimizing fertilization significantly increased the content of soil organic matter and soil carbon storage, and the soil alkaline nitrogen, available phosphorus, and available potassium contents were significantly higher than those under conventional fertilization. The activities of soil urease, sucrase, phosphatase, and catalase were significantly increased under the optimizing fertilization treatment. The changes in the numbers of soil bacteria, actinomycetes, and the total number of soil microorganisms were consistent with enzyme activity. The proportion of soil bacteria to the total number of microorganisms increased, indicating that the combination of organic fertilizer and chemical fertilizer treatment led to the transformation of albic soil from low-fertility (fungal) soil to high-fertility (bacterial) soil. The correlation analysis showed that soil chemical fertility and biological activity significantly contribute to maize yield.

Therefore, optimizing fertilization is of great significance for improving the physical structure of albic soil, enhancing soil fertility levels, and increasing biological activity. In addition, it is one of the important agronomic measures to stabilize and improve crop yields in the region, and will lay the foundation for research on fertility improvement and green fertilization in the albic soil area of China.

## Figures and Tables

**Table 1:** Changes in solid, liquid, and gas phases, and bulk density, under different fertilization treatments.

Treatment	Solid Phase/ %	Liquid Phase/ %	Gas Phase/ %	Bulk Density/ (g·cm^−3^)
T 1	51.85 (±2.64) a	21.41 (±1.84) a	26.74 (±4.45) a	1.198 (±0.122) a
T 2	48.06 (±5.48) a	22.97 (±1.06) a	28.97 (±5.19) a	1.183 (±0.013) a
T 3	47.11 (±3.20) a	22.94 (±2.48) a	29.96 (±5.44) a	1.185 (±0.025) a

Note: T1: conventional fertilization (chemical fertilizer), T2: optimized fertilization 1 (low amount of organic fertilizer + chemical fertilizer), T3: optimized fertilization 2 (high amount of organic fertilizer + chemical fertilizer). Different lowercase letters indicate significant difference among treatments at the *p* < 0.05 level.

**Table 2:** Chemical nutrient contents of albic soil under different fertilization treatments.

Treatments	Alkaline Nitrogen/ (mg·kg^−1^)	Available Phosphorus/ (mg·kg^−1^)	Available Potassium/ (mg·kg^−1^)	pH
T1	204.8 (±5.9) b	23.0 (±1.9) b	106.7 (±2.7) c	6.34 (±0.07) a
T2	213.6 (±3.7) ab	31.9 (±2.0) a	136.4 (±2.5) b	6.35 (±0.05) a
T3	221.5 (±4.9) a	36.5 (±3.5) a	153.2 (±2.9) a	6.39 (±0.06) a

Note: T1: conventional fertilization (chemical fertilizer), T2: optimized fertilization 1 (low amount of organic fertilizer + chemical fertilizer), T3: optimized fertilization 2 (high amount of organic fertilizer + chemical fertilizer). Different lowercase letters indicate significant difference among treatments at the *p* < 0.05 level.

**Table 3:** The proportions of bacteria, fungi, and actinomycetes to total microorganisms in albic soil under different fertilization treatments.

Treatment	Total Number of Microorganisms (×10^5^)	Proportion of Bacteria/%	Proportion of Fungi/%	Proportion of Actinomycetes/%
T1	62.29 b	89.98 a	1.44 a	8.58 a
T2	67.94 b	90.22 a	1.27 ab	8.51 a
T3	80.34 a	90.83 a	1.05 b	8.12 a

Note: T1: conventional fertilization (chemical fertilizer), T2: optimized fertilization 1 (low amount of organic fertilizer + chemical fertilizer), T3: optimized fertilization 2 (high amount of organic fertilizer + chemical fertilizer). Different lowercase letters indicate significant difference among treatments at the *p* < 0.05 level.

## Data Availability

The original contributions presented in this study are included in the article, further inquiries can be directed to the corresponding author.
